# KDM4C-mediated senescence defense is a targetable vulnerability in gastric cancer harboring *TP53* mutations

**DOI:** 10.1186/s13148-023-01579-6

**Published:** 2023-10-17

**Authors:** Kaiqing Wang, Zhicheng Gong, Yanyan Chen, Meimei Zhang, Suzeng Wang, Surui Yao, Zhihui Liu, Zhaohui Huang, Bojian Fei

**Affiliations:** 1 Department of Gastrointestinal Surgery, Affiliated Hospital of Jiangnan University, Wuxi, 214062 Jiangsu China; 2 Wuxi Cancer Institute, Affiliated Hospital of Jiangnan University, Wuxi, 214062 Jiangsu China; 3 Laboratory of Cancer Epigenetics, Wuxi School of Medicine, Jiangnan University, Wuxi, 214122 Jiangsu China; 4 Key Laboratory of Shaanxi Administration of Traditional Chinese Medicine for TCM Compatibility, Shaanxi University of Chinese Medicine, Xi’an, 712046 Shaanxi China

**Keywords:** Gastric cancer, *TP53* mutation, Epigenetic, Senescence, Senolytics

## Abstract

**Background:**

Gastric cancer patients harboring a *TP53* mutation exhibit a more aggressive and chemoresistant phenotype. Unfortunately, efforts to identify the vulnerabilities to overcome these aggressive malignancies have made minimal progress in recent years. Therefore, there is an urgent need to explore the novel therapeutic strategies for this subclass. Histone methylation modulators are critical epigenetic targets for cancer therapies that help maintain the malignancies of cancers harboring *TP53* mutations and senescence evasion. Triggering senescence is now considered to benefit multiple cancer therapies. Furthermore, senescence-based “one-two punch” therapy was validated in clinical trials. Therefore, we hypothesized that screening epigenetic modulators might help identify a novel vulnerability to trigger senescence in gastric cancer harboring *TP53* mutations.

**Results:**

We developed a novel efficient approach to identify senescence inducers by sequentially treating cells with drug candidates and senolytic agents. Based on this, we demonstrated that QC6352 (a selective KDM4C inhibitor) efficiently triggered cellular senescence in gastric cancer harboring *TP53* mutations. More importantly, the “one-two punch’ therapy consisting of QC6352 and SSK1 eliminates tumor cells harboring *TP53* mutations. This finding highlights a potential therapeutic strategy for the aggressive subgroup of gastric cancer. Besides, the functions of QC6352 were totally unknown. We demonstrated that QC6352 might possess far more powerful anti-tumor capacities compared to the traditional genotoxic drugs, 5-Fu and Oxaliplatin.

**Conclusions:**

This initial investigation to identify a senescence inducer revealed that QC6352 triggers senescence in gastric cancer cells harboring *TP53* mutations by regulating the SP1/CDK2 axis through suppressing KDM4C. QC6352 and senolytic agent-SSK1 represent a novel ‘one-two punch’ therapeutic strategy for the more malignant gastric cancer subtypes.

**Supplementary Information:**

The online version contains supplementary material available at 10.1186/s13148-023-01579-6.

## Highlights

- Sequentially treating cells with drug candidates and senolytic agents serve as a novel efficient approach to identify senescence inducers
- QC6352 acts as a novel pro-senescence drug in gastric cancer harboring *TP53* mutations
- The “one-two punch’ therapy consisted of QC6352 and SSK1 eliminates tumor cells harboring *TP53* mutations

**Supplementary Information:**

The online version contains supplementary material available at 10.1186/s13148-023-01579-6.

## Introduction

Gastric cancer remains one of the most common malignancies and lacks an efficient therapeutic strategy [1]. Notably, similar to most solid epithelial cancers, gastric cancer often harbors genomic alterations and *TP53* is the most frequently mutated one in gastric cancer [2]. Many investigations reveal that cancers harboring p53 mutations exhibit a more aggressive and drug-resistant phenotypes [3]. Unfortunately, efforts to identify the vulnerabilities to overcome these aggressive malignancies (including gastric cancer) have made minimal progress in recent years [4].

Mutant p53 promotes evasion of growth arrest and senescence across human cancers [5]. Triggering senescence is now considered to benefit multiple cancer therapies [6]. Inducing cancer cell senescence increases the therapeutic efficacy of immune checkpoint inhibitors by reshaping the immune microenvironment [7]. Furthermore, there is a significant transcriptome change inside senescent cells [8]. These alterations impact fundamental processes such as apoptosis, leading to the acquisition of new vulnerabilities specific to senescent cells that could be selectively cleared by senolytic agents [9]. Importantly, the one-two punch sequential therapy of pro-senescence drugs followed by senolytic therapy is being evaluated in clinical trials [10, 11]. However, it is unknown whether senescence-based combination therapies can be used for *TP53*-mutated tumors and drugs that efficiently induce senescence in such tumors.

Multiple stimuli that induce cellular senescence rely on p53 induction [12, 13]. However, p53 expression levels are often aberrantly high in cells harboring *TP53* mutations [14, 15]. It is intriguing how these cells with *TP53* mutations evade senescence. Notably, histone methylation reprogramming is involved in *TP53* mutation-associated malignancies [16]. Meanwhile, several histone methylation modulators prevent cancer cells from senescence including LSD1, JMJD2C, SUV39H1 and Ash2l [17–19]. Therefore, we hypothesize that histone methylation modification regulators may promote resistance to senescence in *TP53*-mutated tumors. Based on this, we screened a histone demethylase inhibitor library by sequentially treating p53-mutated cells with histone demethylase inhibitors and SSK1 (a senolytic agent) and revealed a pivotal role of targeting KDM4C in triggering senescence in cells harboring p53 mutations by regulating SP1/CDK2 signaling. These data provide a novel therapeutic strategy for the aggressive and chemoresistant gastric cancer harboring *TP53* mutations.

## Materials and methods

### Cell culture

Human gastric cancer cell lines, NCI-N87, HGC27, and AGS, were purchased from ATCC and were cultured in Dulbecco’s modified Eagle’s medium (DMEM) (Gibco) supplemented with 10% fetal bovine serum (FBS) (EallBio, # 03.U16001). All cells were maintained at 37 °C in a saturated humidity atmosphere containing 95% air and 5% CO_2_

### Immunoblotting

Immunoblotting analyses involved lysing cells or tissue samples in RIPA lysis buffer (Invitrogen, #89900). The protein concentration of each sample was assessed using the bicinchoninic acid (BCA) kit (Yeasen, #20201ES86) according to the manufacturer’s instructions. Equal amounts of protein extracts were separated by electrophoresis on appropriate Tris-Glycine gels (Yeasen, #36252ES10) and subsequently transferred to polyvinylidene difluoride (PVDF) membranes (Millipore, #IPVH00010). The membrane was probed with primary antibodies listed below, followed by secondary antibodies conjugated to horse radish peroxidase (HRP). The immunoblot bands were analyzed using Image Lab software.

### Cell counting kit-8 (CCK-8) assay

Cells were dispensed into 96-well plates after indicated treatments and incubated with the medium containing 10% Cell Counting Kit-8 reagent (ApexBio, #K1018) for 2 h at 37 °C. The OD450 nm value was measured using a microplate reader.

### Pro-senescence drug screening

Cells were seeded onto 96-well plates at a density of 3000 cells per well followed by sequentially treating with individual inhibitors from the Epigenetics Compound Library (MCE, #HY-L005, 10 nM) and dimethyl sulfoxide (DMSO) or SSK1 (MCE, #HY-138936, 1 μM) for 96 h. Cell viability was determined using a CCK-8 assay, and the data were visualized by GraphPad Prism 8.0.

### Senescence-associated β-galactosidase (SA-β-gal) analysis

Senescence-associated expression of β-gal activity was determined by a Senescence Detection kit (Solarbio, #G1580-100T) according to the manufacturer’s instructions. Briefly, cells were seeded onto 6-well plates at a density of 1 × 10^5^ and treated with DMSO or gradient doses of QC6352 (20 nM) for 96 h. Subsequently, cells were fixed by adding 1 mL β-Gal Fixative buffer at room temperature for 15 min followed by staining with Dye Working Solution at 37 °C overnight.

### Immunofluorescence

Cells were seeded on a 6-well plate with coverslips, followed by treatment with QC6352 (20 nM) for 96 h. The culture medium was removed, and coverslips were carefully washed three times with phosphate-buffered saline (PBS). The cells were fixed by incubating in 4% paraformaldehyde for 5 min at room temperature, washing twice with PBS and twice with washing buffer. Cells were then permeabilized with 0.5% Triton X-100 for 5 min, blocked in PBS containing 1% bovine serum albumin (BSA), and subsequently incubated with primary antibodies against H3K9me3 (CST, #9649S) at 4 °C overnight. Cells were washed three times with PBS and then incubated with Alexa Fluor 488 goat anti-rabbit (Life Technologies) at room temperature for 1 h in the dark. The nuclei were extensively washed with ice-cold PBS and then counterstained with 4’,6-diamidino-2-phenylindole (DAPI) for 10 min. Specimens were mounted in 70% glycerol and sealed with nail polish. Fluorescent images were taken using an Olympus fluorescence microscope.

### Cell cycle analysis

Cells treated with QC6352 were collected and washed with PBS, fixed with 70% ethanol, and resuspended in PBS containing propidium iodide and RNase A (200 µg/mL). Cell cycle analyses were performed using a NovoCyte flow cytometer and FlowJo software v10 (Treestar, Ashland, USA).

### Enzyme linked immunosorbent assay (ELISA)

Cell-cultured medium was centrifuged, and the supernatant was subjected to ELISA detection according to the manufacturer’s instructions of the Human IL-6 ELISA Kit (BOSTER, #EK0410) and IL-8 Elisa Kit (BOSTER, #EK0413).

For tumor tissues, 0.1 g of tissue blocks were first transferred into a glass homogenizer containing 1 mL of pre-cooled PBS and 10 μL 100mM phenylmethylsulfonyl fluoride (PMSF), followed by thorough grinding and ultrasonication on ice. The prepared homogenate was centrifuged at 5000 × g for 5 min, and the supernatant was collected for ELISA assays.

### 7-amino-actinomycin D (7-AAD) staining

Gastric cancer cells (2 × 10^5^ cells/well) were seeded in 6-well plates, treated with DMSO or QC6352 (20 nM), followed by DMSO or SSK (1 μM) treatment. Subsequently, cells were collected and washed with PBS. After centrifugation, the cell pellets were resuspended and then incubated with 50 μL 1 × Assay Buffer containing 5 μL 7-AAD Solution at room temperature for 15 min, followed by mixing with 450 μL 1×Assay Buffer working solution. After that, the death cell rates (positive 7-AAD cells) were assessed by NovoCyte flow cytometer.

### Animal studies

Male nude mice (BALB/C, 15–20 g, 4–6 weeks old) were obtained from SPF (Beijing) Biotechnology and maintained under pathogen-free conditions. A total of 3 × 10^6^ NCI-N87 cells resuspended in a 1:1 solution of PBS and Matrigel Matrix (Corning) were subcutaneously injected into the dorsal flanks of mice.

The mice were numbered and randomized into vehicle, 10 mg/kg QC6352, and / or 1 mg/kg SSK1 treatment groups when tumor volume reached approximately 13.5 mm^3^. Here, QC6352 and SSK1 were resolved and suspended in 10% DMSO (MCE, #HY-Y0320) and 90% corn oil (MCE, #HY-Y1888).

Mice weight and tumor volume was recorded daily by caliper measurements using the following formula: π (width × length) / 6 (mm^3^). Mice were sacrificed when the tumor size in the vehicle-treated group exceeded 1000 mm^3^ (21 days after treatment). The tumors were dissected, weighed, and photographed.

### Bioinformatic analysis

RNA-sequencing expression (level 3) profiles and corresponding clinical information for gastric cancer were downloaded from The Cancer Genome Atlas (TCGA) dataset (https://portal.gdc.com). The two-gene correlation map is realized by R software package ggstatsplot, and the multi-gene correlation pheatmap is displayed by R software package. Spearman’s correlation analysis was used to describe the correlation between quantitative variables without a normal distribution. *P* values less than 0.05 were considered statistically significant.

The ATF6, SP1, and KDM4C expression levels in normal and gastric cancer tissues were determined by the online tool GEPIA2 (http://gepia2.cancer-pku.cn/).

Kaplan–Meier survival curves were generated using the Kaplan–Meier Plotter website for gastric cancer (Version 2020, http://kmplot.com) and statistical significance was determined by the log-rank test.

The correlation between candidate genes and age was obtained from UALCAN (http://ualcan.path.uab.edu)

### Colony formation assay

Cells were cultured in 6-well plates at a density of 3000 cells per well, followed by treatment with the indicated compounds for 14 days and staining with crystal violet (Beyotime, #C0121). The colonies were analyzed by ImageJ software.

### Transwell assay

In vitro migration assays used an 8 µm pore size Boyden chamber (Corning Costar). Cells (200 µL, 1 × 10^5^) in serum-free DMEM were plated in the upper chamber, and 500 µL 10% FBS was added to DMEM in the lower chamber as a chemoattractant. After 12 h, cells on the upper side of the filter were removed and cells that remained adherent to the underside of membranes were fixed in methanol, followed by staining with 0.1% crystal violet. The number of migrated cells was counted using a microscope. Five contiguous fields of each sample were examined using a 20 × objective to obtain a representative number of cells that migrated across the membrane.

### Study approval

Prior to obtaining patient samples, requisite approval from the Medical Ethics Committee of the affiliated hospital of Jiangnan University and written informed consent from patients were obtained. The mouse experiments were approved by Institutional Animal Care and Use Committee of the affiliated hospital of Jiangnan University.

### Statistical analyses

All statistical analyses were performed using GraphPad Prism 8.0 (GraphPad Software, USA). Differences between two groups were analyzed using an unpaired Student’s t test, while differences among multiple groups were analyzed by one-way analysis of variance (ANOVA). Results were considered statistically significant when *p* < 0.05.

## Results

### Histone demethylase inhibitor library screening identifies QC6352 as a potent pro-senescence drug in cells harboring p53 mutations

**Fig. 1:**
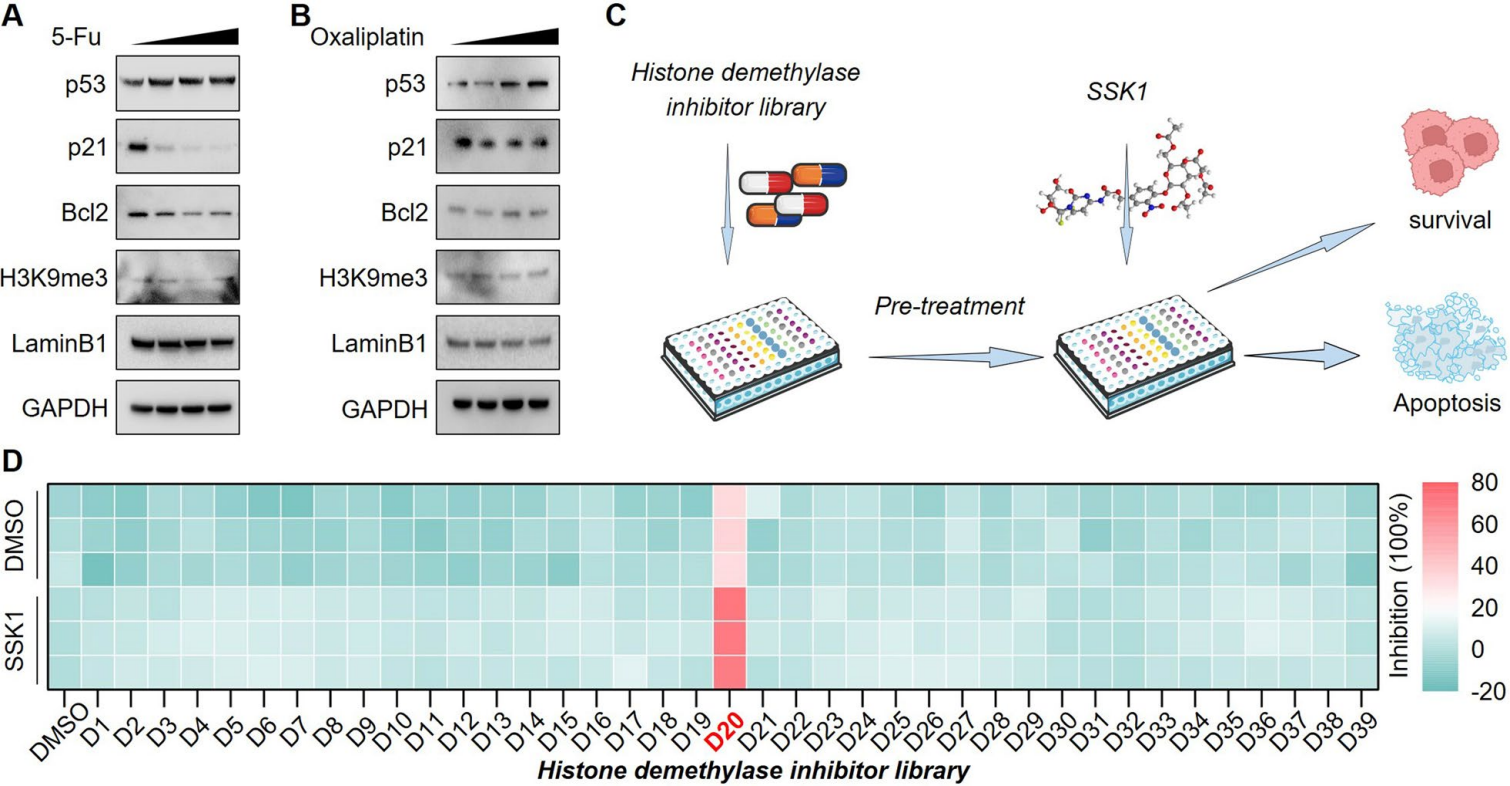
QC6352 served as a potential pro-senescence agent in gastric cancer harboring TP53 mutations **A-B** NCI-N87 was treated with low dose genotoxic agents, followed by immunoblotting analysis as indicated. **C** Flow chart of the novel screening strategy for pro-senescence drugs. **D** Cells were seeded onto 96-well plates, followed by sequential treatment with individual inhibitors from the Epigenetics Compound Library (10 nM) and DMSO or SSK1 (1 μM). After 96 h, the cell viability was determined by Cell Counting Kit-8 (CCK-8) assays and the heatmap summarized cell viabilities under the indicated treatment. The details of related compounds in this figure are listed in Additional file 1: Table s1

R248 is one of the most common mutation sites occurring in the DNA binding domain of p53 that results in the inability of p53 to bind to DNA [20]. NCI-N87 cells (a cell line derived in 1976 from the stomach of a male gastric carcinoma patient) carry the p53^R248^ mutation. We first assessed the IC_50_ values of the genotoxic agents, 5-Fu and Oxaliplatin, in this cell line (Additional file 2: Figure S1A-B). Low doses of chemotherapy triggered senescence instead of apoptosis [6]. Therefore, we treated the NCI-N87 cells with gradient dose below the IC_50_ of 5-Fu or Oxaliplatin. These doses induced p53 expression, although pro-senescence downstream p21 was still inactivated (Fig. 1A, B). These data suggested that NCI-N87 cells were a suitable model to identify senescence inducers in cells harboring *TP53* mutations. Inspired by the one-two punch therapy, we sequentially treated NCI-N87 cells with the indicated compounds from the inhibitor library and a previous reported senolytic agent, SSK1 [21] (Fig. 1C). Interestingly, SSK1 alone was insufficient to induce cell death, but eliminated the cancer cells pre-treated with KDM4C selective inhibitor (QC6352) (Fig. 1D). Taken together, these data indicate that QC6352 might be a novel pro-senescence compound in p53-mutated cancer cells.

### QC6352 acts as a novel pro-senescence drug

**Fig. 2:**
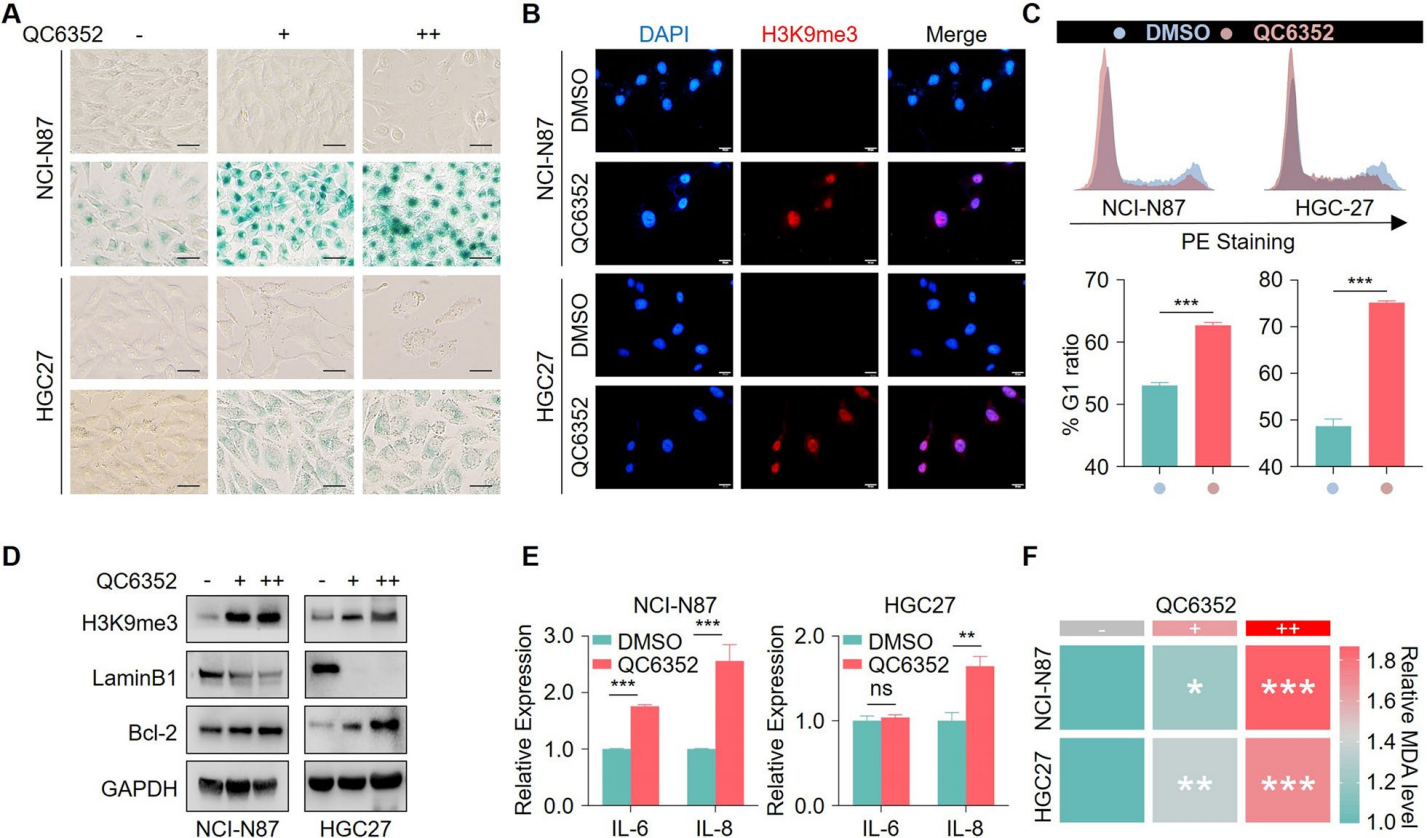
QC6352 triggered senescence in gastric cancer harboring the TP53 mutation. **A** Representative images of SA-β-Gal staining in gastric cancer cells treated as indicated. Scale bar, 20 μm. **B** Immunofluorescence staining for H3K9me3 in cells before and after QC6352 (20 nM) treatment. Scale bar, 20 μm. **C** Gastric cancer cells were treated with DMSO or QC6352 (20 nM) and the cell cycle profile was obtained by propidium iodide (PI) staining and fluorescent-activated cell sorting (FACS) analysis. **D** Gastric cancer cells were treated with gradient doses of QC6352 (10 nM and 20 nM), followed by immunoblotting. **E** Gastric cancer cells were treated with 20 nM QC6352, followed by enzyme linked immunosorbent (ELISA). **F** Gastric cancer cells were treated with gradient doses of QC6352 (10 nM and 20 nM), followed by malondialdehyde (MDA) detection. *P < 0.05, **P < 0.01, and ***P < 0.001. *P* values were determined by two-tailed unpaired t test. Data were presented as mean ± SEM of three independent experiments

The increase of SA-β-gal, a robust induction of H3K9me3, stable G1 arrest, loss of Lamin B1, activation of anti-apoptotic protein Bcl-2, secretion of pro-inflammatory cytokines, and oxidant stress accumulation are defined as the hallmarks of senescent cells [6]. The pro-senescence capacity of QC6352 was validated using all of these senescent markers in two p53-mutated cells (NCI-N87 and HGC-27) following QC6352 treatment. Initially, we confirmed that p53 expression was undetected owing to HGC-27 cells harboring a frameshifted *TP53* and the low dose of genotoxic agents could not induce p21 expression in HGC-27 cells (Additional file 2: Figure S2A-D). Consistent with our hypothesis, we observed that the cell sizes of these two cell lines were enlarged accompanied with positive SA-β-gal staining after QC6352 treatment (Fig. 2A). A robust accumulation of H3K9me3 occurred in these cells upon QC6352 treatment (Fig. 2B). Furthermore, cell cycle distribution of these senescence-like cells unsurprisingly found that QC6352 treatment led to a significant G1 arrest in both *TP53* mutated cell lines (Fig. 2C). Moreover, immunoblotting analysis confirmed the remarkable induction of H3K9me3m and revealed that QC6352 treatment led to a loss of LaminB1 and a robust increase of BCL-2 (Fig. 2D). Meanwhile, the secretion of pro-inflammatory cytokines also increased upon QC6352 treatment in these cells (Fig. 2E). Besides, QC6352 treatment led to a significant accumulation of oxidative stress as evidenced by the drastically increased malondialdehyde (MDA) levels upon QC6352 treatment (Fig. 2F). Taken together, these data demonstrate that QC6352 acted as a novel pro-senescence drug in cells harboring p53 mutations.

### SSK1 efficiently eliminates QC6352-induced senescent cells

**Fig. 3:**
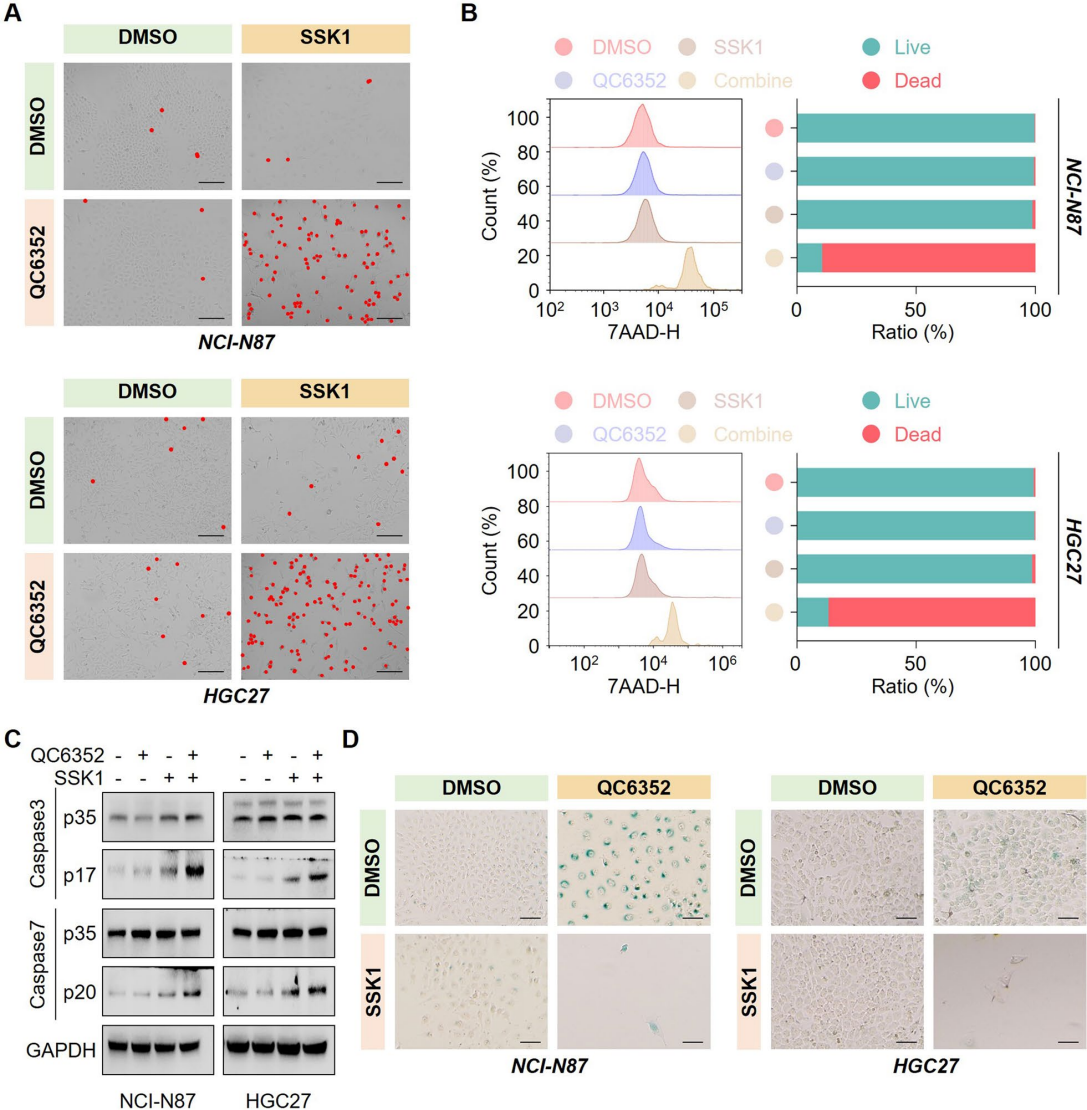
SSK1 efficiently eliminated QC6352-induced senescent cells. **A-B** Representative images of cell death (**A**), quantification of cell death by 7-amino-actinomycin D (7AAD) staining (**B**) in NCI-N87 and HGC27 cells treated with QC6352 for 96 h in the presence or absence of SSK1. **C** NCI-N87 and HGC27 cells were sequentially treated with QC6352 and SSK1, followed by immunoblotting analysis. **D** Representative images of senescence-associated β-galactosidase (SA-β-Gal) staining in gastric cancer cells sequentially treated as indicated. Scale bar, 20 μm

Senescent cells acquire new vulnerabilities to senolytic agents like SSK1 [9, 21]. These senolytic agents were used in clinical trials across human cancers in combination with (or sequentially with) senescence-inducing therapies [6]. Hence, we explored whether SSK1 could efficiently clear QC6352-induced senescent cells. Unsurprisingly, extensive cell death occurred in cells sequentially treated with QC6352 and SSK1 (Fig. 3A). This observation was confirmed by 7-AAD staining (Fig. 3B). The activation status of Caspase 3 and Caspase 7 apoptosis effectors was significantly activated in cells treated with our novel “one-two punch” combination (Fig. 3C). Moreover, SSK1 selectively eliminated senescent cells in our system since the percentage of SA-β-gal positive cells dramatically reduced when sequentially treated with QC6352 and SSK1 (Fig. 3D). Collectively, these data indicated QC6352 in combination with SSK1 could act as a novel “one-two punch” for cancer therapy in vitro.

### QC6352 in combination with SSK1 inhibited tumor growth in vivo

**Fig. 4:**
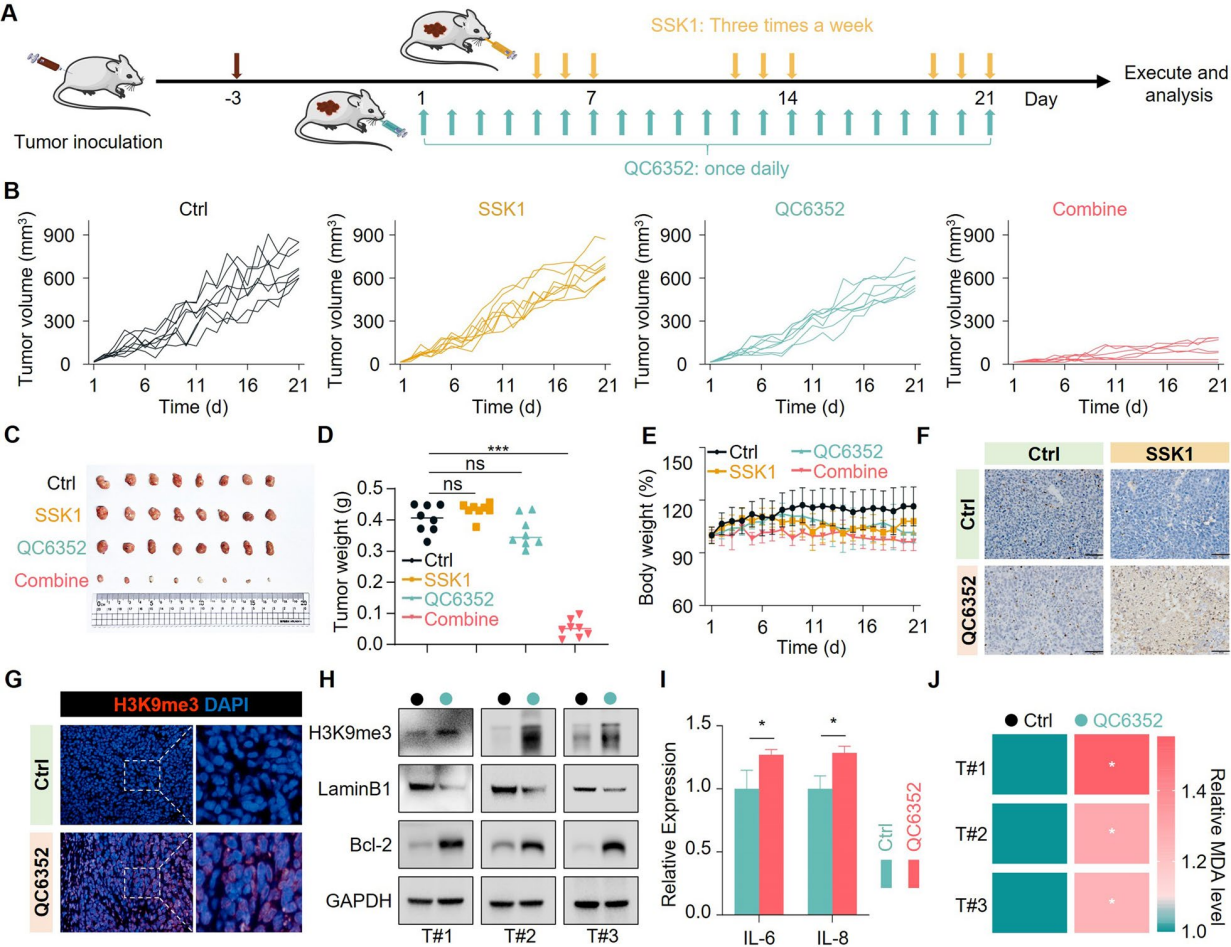
QC6352 combined with SSK1 inhibited tumor growth *in vivo.* **A** The workflow of the “one-two punch” therapeutic mouse model. **B** Spider plots of tumor growth in each group. Each line represented one mouse (n = 8). **C** Representative images of xenograft tumors with the indicated treatment at the end point (n = 8). **D** Scatter plot of xenograft tumors with the indicated treatment at the end point (n = 8). **E** The body weight of mice in each group was recorded daily and summarized as indicated (n = 8). **F** Representative terminal deoxynucleotidyl transferase dUTP nick end labeling (TUNEL) staining in tumor tissues derived from the indicated group. Scale bar, 50 μM. **G** Representative immunofluorescence staining for H3K9me3 in tumor tissues derived from the indicated group. Scale bar, 50 μM. **H** The tumor tissues derived from each group were lysed, followed by immunoblotting analysis (n = 3). **I** The tumor tissues derived from each group were lysed, followed by ELISA analysis (n = 3). **J** MDA levels in tissues derived from the indicated group were determined by an MDA kit. **P < 0.01 and ***P < 0.001 from two-tailed unpaired Student’s t tests. Data represent the mean ± SD (n = 8 mice per group)

We constructed a tumor xenograft mouse model and treated it with our novel “one-two punch” combination to examine the anti-cancer effects in vivo (Fig. 4A). QC6352 or SSK1 treatment alone merely affected the tumor growth whereas QC6352 combined with SSK1 dramatically suppressed tumor growth *in vivo* (Fig. 4B-D). This was consistent with the in vitro results. Notably, this novel therapeutic strategy exerted a powerful anti-tumor effect without affecting the body weight of mice (Fig. 4E). Terminal deoxynucleotidyl transferase dUTP nick end labeling (TUNEL) staining further supported that QC6352 combined with SSK1 triggered cell death to suppress tumor progression *in vivo* (Fig. 4F). The pro-senescent role of QC6352 *in vivo* was confirmed by staining the xenografted tumor tissues with H3K9me3 since the H3K9me3 positive rates in mice treated with QC6352 were much higher compared to the control group (Fig. 4G). Meanwhile, a consistent alteration of senescence markers was observed in the *in vitro* model and in xenografted tumor tissues. This further supported that QC6352 efficiently triggered senescence in *TP53*-mutated tumors (Fig. 4H). In addition, the secretion of senescence-related pro-inflammatory cytokines was also induced in QC6352-treated tumors (Fig. 4I). Consistently, the MDA levels in tissues derived from QC6352 treated tumors were much higher than that in tissues separated from the control group (Fig. 4J). In conclusion, QC6352 can be combined with senolytic agents to form a novel “one-two punch” strategy for cancer therapy.

### QC6352 triggers senescence by potentially repressing SP1-CDK2 signaling

**Fig. 5:**
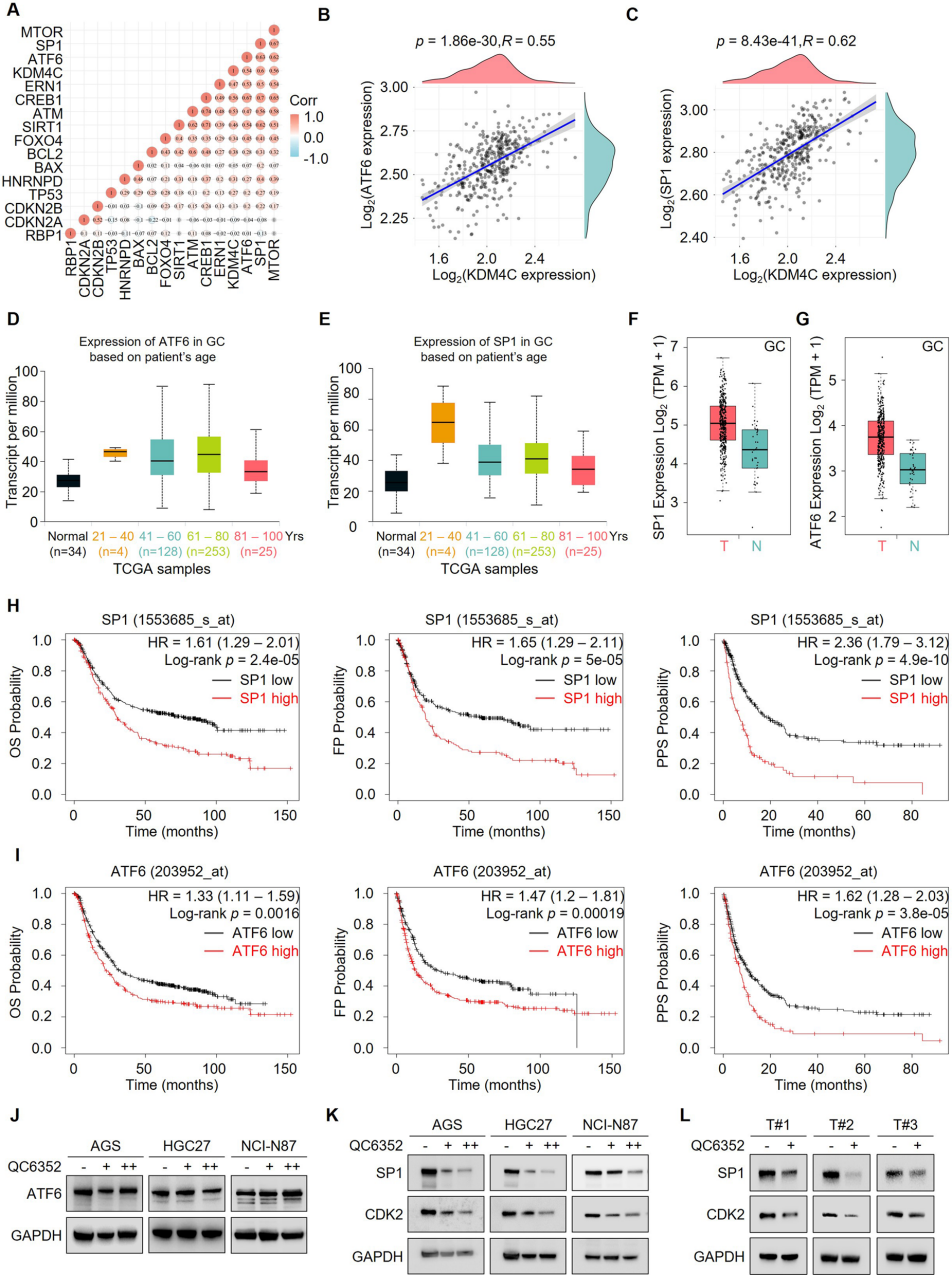
QC6352 triggered senescence by potentially repressing SP1-CDK2 signaling. **A** Triangular heatmap representing the pairwise correlation among KDM4C and the key pro-senescence genes. **B-C** Expression correlation between KDM4C with ATF6 (**B**) and SP1 (**C**). **D-E** The Cancer Genome Atlas (TCGA) RNA-sequencing results showing the expression levels of ATF6 (**D**) or SP1 (**E**) in the indicated type of patients with gastric cancer. **F-G** The RNA expression of SP1 (F) and ATF6 (G) in a gastric cancer cohort of TCGA. **H-I** Kaplan–Meier plots analysis of overall survival (OS), first progression (FP), and post-progression survival (PPS) rates in patients with gastric cancer with high or low SP1 mRNA levels (H), or high or low ATF6 mRNA levels (I). **J-K** Gastric cancer cells were treated with gradient doses of QC6352 (10 nM and 20 nM), followed by immunoblotting analysis.** L** Tumor samples derived from the indicated groups were lysed, followed by immunoblotting analysis

QC6352 is a selective inhibitor of KDM4C. Meanwhile, KDM4C governs genes transcription via demethylating H3K36me3 or H3K9me3. Hence, we screened the correlation between KDM4C and senescence-associated genes to gain an insight into the potential downstream targets that mediated the pro-senescence function of KDM4C. Four genes were significantly positively correlated with KDM4C (Fig. 5A). Only genes whose expression downregulation could drive senescence were selected for further studies. We further confirmed the positive correlation between these two genes and KDM4C (Fig. 5B, C). Interestingly, ATF6 and SP1 RNA transcripts negatively correlated with ages in patients with gastric cancer (Fig. 5D, E). Notably, these two genes were aberrantly upregulated in tumor tissues compared to the corresponding normal tissues (Fig. 5F, G). Besides, gastric cancer patients with higher expression of either ATF6 or SP1 had poor survival rates which suggested that these genes might act as a potential biomarker for diagnosis (Fig. 5H, I). More importantly, assessment of ATF6 and SP1 expression in cells treated with QC6352 found that only SP1 and its downstream target-CDK2 (which was known to mediate senescence induced by SP1 depletion) were significantly downregulated upon QC6352 treatment (Fig. 5J, K). More importantly, a remarkable downregulation of SP1-CDK2 signaling was observed in tumors treated with QC6352 (Fig. 5L). Collectively, these results highlight that QC6352 might trigger cellular senescence by downregulating SP1-CDK2 signaling through inhibiting KDM4C.

### QC6352 possessed strong anti-cancer activity in vitro

**Fig. 6:**
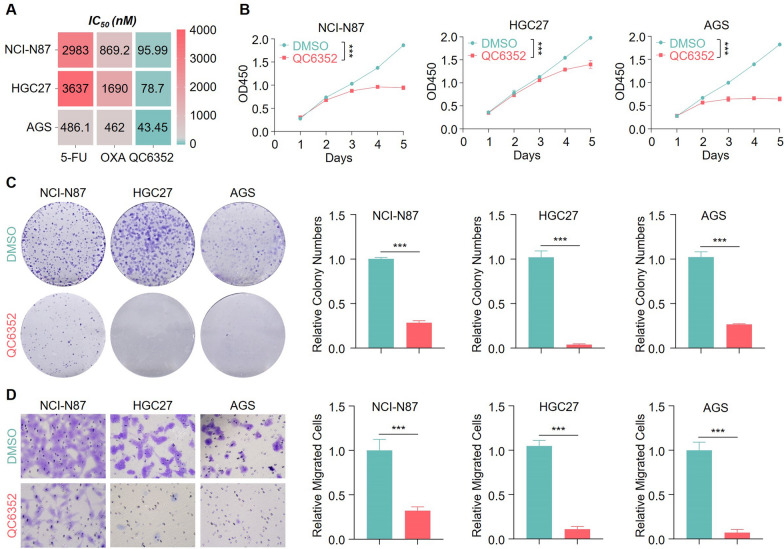
QC6352 possessed strong anti-cancer activity. **A** Bar plot representing the IC_50_ of genotoxic agents and QC6352 in the indicated cell lines. **B-D** NCI-N87, HGC27, and AGS cells were treated with QC6352 (96 nM for NCI-N87, 79 nM for HGC27 and 44 nM for AGS) and then subjected to cell proliferation assays (**B**), colony formation assays (**C**) and Transwell migration assays (**D**). ***P < 0.001. *P* values were determined by a two-tailed unpaired t test. Data were presented as mean ± SEM of three independent experiments

The role of QC6352 in gastric cancer was totally unknown. Therefore, we explored whether QC6352 administration alone exerted anti-tumor functions other than triggering senescence when increasing its dosage. We initially assessed the IC_50_ of QC6352 in gastric cancer cell lines and found that the anti-tumor activity of QC6352 far exceeded that of conventional chemotherapy drugs (Fig. 6A). Treatment of three independent gastric cancer cell lines with their corresponded IC_50_ dose of QC6352 showed that QC6352 alone dramatically suppressed gastric cancer cell proliferation and colony formation capacities with increasing dosage (Fig. 6B, C). In addition, high dosages of QC6352 alone also exhibited strong anti-metastasis activity *in vitro* (Fig. 6D). In conclusion, these data enriched the knowledge of the function of QC6352 and highlighted its potential clinical therapeutic value.

### KDM4C serves as a potential biomarker for diagnosis in gastric cancer

**Fig. 7:**
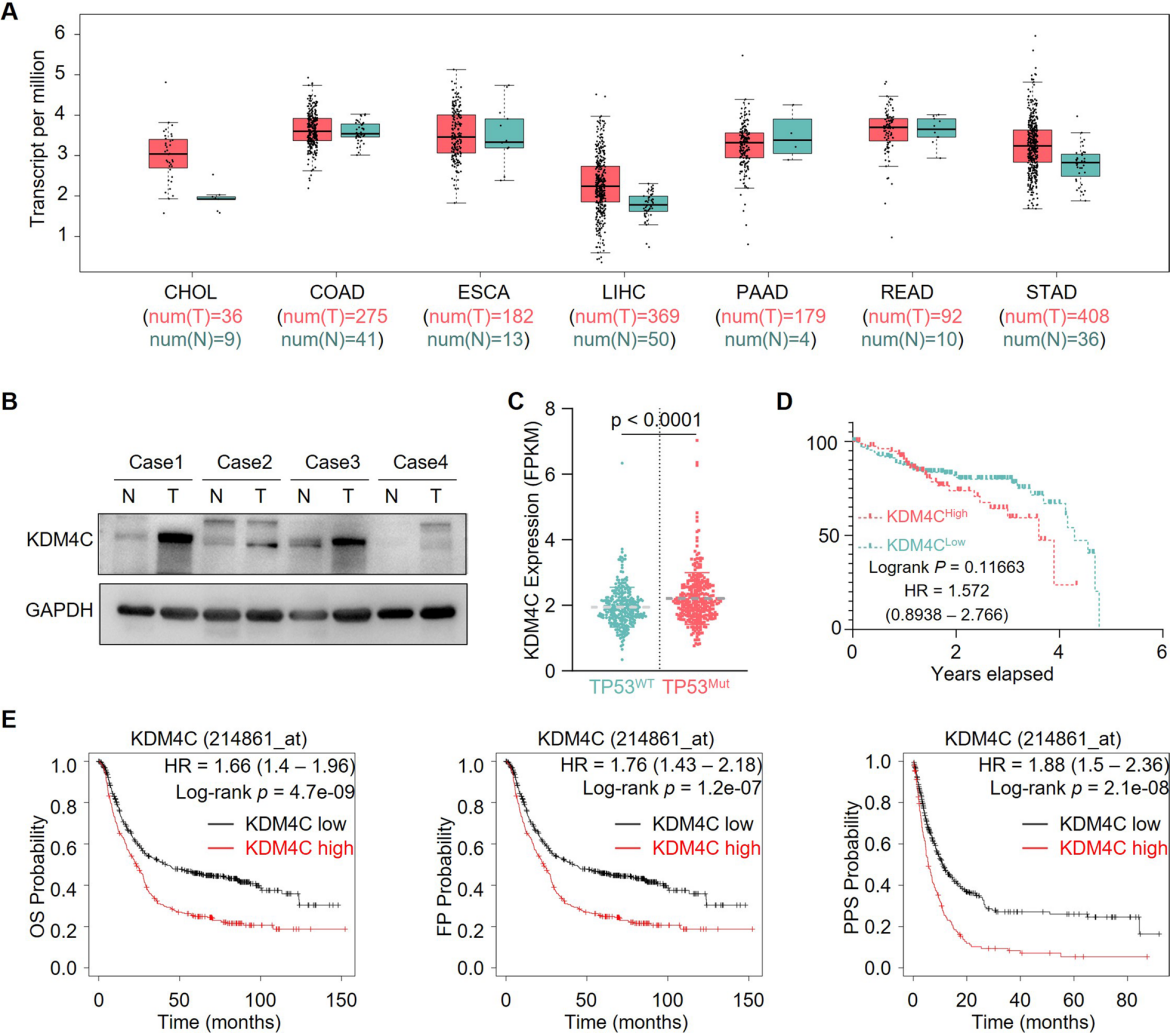
KDM4C served as a potential biomarker in gastric cancer. **A**, The expression of KDM4C in the digestive system cancer cohort of TCGA. **B** Four paired tissues from patients with gastric cancer were lysed, followed by immunoblotting analysis. **C** The RNA expression of KDM4C in the gastric cancer cohort of TCGA. **D** Kaplan–Meier plots analysis of OS in TP53-mutated gastric cancer patients with high or low KDM4C mRNA levels. **E** Kaplan–Meier plots analysis of OS, FP, and PPS in patients with gastric cancer with high or low KDM4C mRNA levels

We explored the clinical implications of QC6352 on its direct target-KDM4C. Increased *KDM4C* transcripts were found in gastric and liver cancer patients based on TCGA database (Fig. 7A). Consistently, immunoblotting detected a profoundly elevated protein level of KDM4C in gastric cancer from our cohort (Fig. 7B). Notably, KDM4C expression levels were drastically higher in gastric cancer harboring *TP53* mutations compared to the wild-type cohort (Fig. 7C). Furthermore, high KDM4C expression levels predicted the poor clinical outcomes in patients with gastric cancer harboring *TP53* mutations (Fig. 7D). Besides, high *KDM4C* transcripts levels predicted the poor outcomes in patients with gastric cancer (Fig. 7E). Collectively, these data suggest that KDM4C might act as a potential biomarker for diagnosing gastric cancer clinical outcomes and further highlights the clinical therapeutic values of QC6352.

## Discussion

Although some precise treatment strategies have been used for the clinical management of gastric cancer, conventional chemotherapy remains the mainstay of treatment [1]. Noteworthy, ~50% of gastric cancers harbor TP53 mutations and these portions often exhibit a more malignant phenotype and are resistant to traditional chemotherapy [2]. Effective therapeutic strategies in this subtype of gastric cancers remain largely unknown. This study identified a novel, highly effective senescence-inducer and proposed a ‘one-two punch’ approach for gastric cancer harboring *TP53* mutations from epigenetic drugs.

Senescence-based ‘one-two punch’ treatments are regarded as one of the effective therapeutic strategies for human cancers [6, 10]. Besides, triggering senescence reprograms the tumor immune-microenvironment to enhance the therapeutic efficacies of immune check point inhibitors [7]. Notably, multiple stimuli including genotoxic drugs drive senescence relying on the induction of p53 and activation of its downstream signaling [6, 12–14]. Intriguingly, p53 expression levels are usually aberrantly high in cancer cells harboring *TP53* mutation in contrast to the p53 wild types [14, 15]. However, these TP53-mutated cancer cells often exhibit a fast growth phenotype instead of growth arrest or senescence [5]. Therefore, we speculate that a certain transcriptional regulator may contribute to this phenomenon. Noteworthy, histone methylation governs multiple gene expression and its alteration are involved in *TP53* mutation-associated malignancies [16]. Besides, several histone methylation modulators prevent cancer cells from senescence [17–19]. Therefore, we speculate that cancer cells harboring *TP53* mutations may evade senescence by reprogramming histone methylation codes. Fortunately, our data deciphered that pharmaceutical blockage of H3K9me3 demethylase (KDM4C) is vulnerable to triggering senescence in gastric cancer cells carrying TP53 mutations.

The KDM4C inhibitor (QC6352) was first designed and validated in 2017 [22]. However, its anti-tumor function was only investigated in breast cancer cells [23]. It is unknown whether it can work on other human cancers. KDM4C contributes to tumor progression across human cancers, suggesting that it may serve as a promising therapeutic target for cancers [24–28]. Notably, little is known about the role of KDM4C in gastric cancer. Only Lang et al. [26] reported that KDM4C depletion inhibits stemness, tumorigenesis, and chemoresistance in gastric cancer stem cells. Here, we revealed that pharmaceutical blockage of KDM4C by QC6352 efficiently triggers senescence in gastric cancer cells harboring a *TP53* mutation. Moreover, we deciphered that QC6352 achieves its senescence-inducing role by regulating the SP1/CDK2 axis. Moreover, we demonstrated that QC6352 possesses a stronger *in vitro* anti-tumor function in gastric cancer cells compared to traditional genotoxic drugs like 5-Fu and Oxaliplatin.

## Conclusion

A novel strategy identified that QC6352 efficiently triggers senescence in gastric cancer cells harboring *TP53* mutations by regulating the SP1/CDK2 axis through suppressing KDM4C. Furthermore, QC6352 and senolytic agent-SSK1 can consist of a novel ‘one-two punch’ therapeutic strategy for the more malignant gastric cancer subtypes.

### Supplementary Information

**Additional file 1. Table S1.** Details of the composition of the histone demethylase inhibitor library.

**Additional file 2. Fig S1.** IC50 determination of genotoxic agents in N87 cells. **Fig S2.** Low doses of genotoxic agents fail to active p53 signaling and trigger senescence in HGC27.

## Data Availability

The data used to support the findings of this study are available from the corresponding author upon request.

## Abbreviations

7-AAD: 7-Amino-actinomycin D
ANOVA: Analysis of variance
β-Gal: Beta galactosidase
BCA: Bicinchoninic acid
BSA: Bovine serum albumin
CCK-8: Cell Counting Kit-8
DAPI: 4′,6-Diamidino-2-phenylindole
DMEM: Dulbecco’s modified Eagle’s medium
DMSO: Dimethyl sulfoxide
ELISA: Enzyme linked immunosorbent assay
FACS: Fluorescent-activated cell sorting
FBS: Fetal bovine serum
HRP: Horse radish peroxidase
MDA: Malondialdehyde
OS: Overall survival
PBS: Phosphate-buffered saline
PFS: Progress free survival
PI: Propidium iodide
PMSF: Phenylmethylsulfonyl fluoride
PPS: Post-progression survival
PVDF: Polyvinylidene difluoride
SA-β-gal: Senescence-associated β-galactosidase
TCGA: The Cancer Genome Atlas
TUNEL: Terminal deoxynucleotidyl transferase dUTP nick end labeling
