# Supplementary material for: KDM4C-mediated senescence defense is a targetable vulnerability in gastric cancer harboring TP53 mutations
Source: Clin Epigenetics. 2023 Oct 17;15:163. doi: 10.1186/s13148-023-01579-6 (PMC10583429; doi:10.1186/s13148-023-01579-6)
Supplement: Supplementary file 2 — Additional file 2. Fig S1. IC50 determination of genotoxic agents in N87 cells. Fig S2. Low doses of genotoxic agents fail to active p53 signaling and trigger senescence in HGC27. [file 13148_2023_1579_MOESM2_ESM.docx]

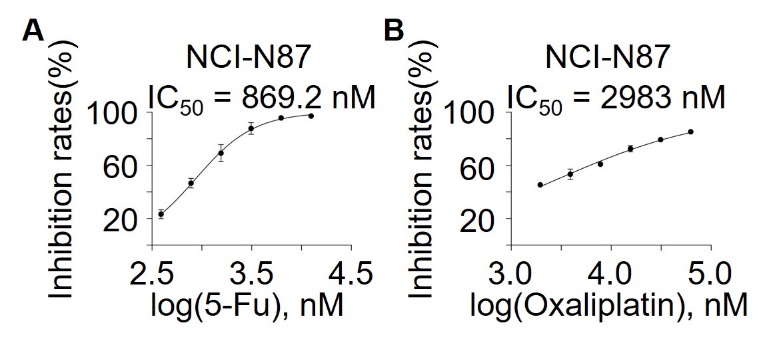


**Supplementary Fig. 1**

**IC50 determination of genotoxic agents in N87 cells**

**A-B**, Dose-response assay in NCI-N87 cells was used to determine the IC_50_ of indicated genetoxic drug.


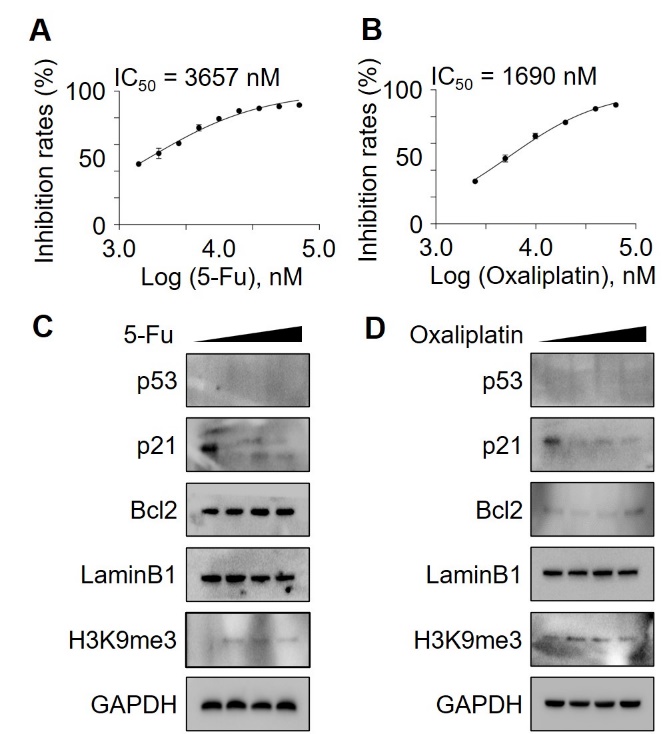


**Supplementary Fig. 2**

**Low doses of genotoxic agents fail to active p53 signaling and trigger senescence in HGC27**

**A-B,** Dose-response assay in HGC27 cells was used to determine the IC_50_ of indicated genetoxic drug. **C-D,** HGC27 cells were treated with low dose of indicated genotoxic drug, followed by IB analysis as indicated.
